# Re-Visiting Maximal Heart Rate Prediction Using Cross-Validation in Population Aged 7–55 Years

**DOI:** 10.3390/ijerph19148509

**Published:** 2022-07-12

**Authors:** Jeong-Hui Park, Hyun Chul Jung, Yeon-Sung Jung, Jong-Kook Song, Jung-Min Lee

**Affiliations:** 1 Department of Physical Education, Kyung Hee University (Global Campus), 1732 Deokyoungdaero, Giheung-gu, Yongin-si 17014, Gyeonggi-do, Korea; jeonghee@khu.ac.kr; 2 Department of Coaching, Kyung Hee University (Global Campus), 1732 Deokyoungdae-ro, Giheung-gu, Yongin-si 17014, Gyeonggi-do, Korea; jhc@khu.ac.kr; 3 Center for Sports Science in Gyeonggi, 134 Jangan-ro, Jangan-gu, Suwon-si 16312, Gyeonggi-do, Korea; doldol11@ggsc.or.kr; 4 Divison of Sport Medicine, Graduate School of Physical Education, Kyung Hee University (Global Campus), 1732 Deokyoungdae-ro, Giheung-gu, Yongin-si 17014, Gyeonggi-do, Korea; jksong@khu.ac.kr; 5 Sports Science Research Center, Kyung Hee University (Global Campus), 1732 Deokyoungdaero, Giheung-gu, Yongin-si 17014, Gyeonggi-do, Korea

**Keywords:** maximal heart rate, maximal heart rate prediction, graded exercise test

## Abstract

The primary purpose of the present study was to re-visit HR_max_ prediction by two commonly used equations (i.e., Fox′s and Tanaka′s equation) compared to the direct measured HR_max_ using the large sample size of Asians. The second aim of the study was to focus on suggesting new equations for the Asian population by separating gender and specific age groups. A total of 672 participants aged from 7 to 55 years were recruited for the study (male: 280 and female: 392), and the maximal graded exercise test with Bruce protocol was used to measure HR_max_. All data obtained from the study were analyzed by SPSS 25.0. Additionally, three statistical analysis methods (i.e., Mean Absolute Percent Errors (MAPE), Bland–Altman plots, and equivalence testing) were utilized to confirm the consistency between the measured HR_max_ and the two prediction equations. The main finding was that two equations showed significant differences in predicting the HR_max_ of Korean aged from 7 to 55 years. The outcome of children aged from 7 to 14 was a different fit in the agreement compared to other age groups. Fox′s equation had the best fit in the average of the difference closer to zero and completely included within the equivalence zone, but females over 15 years old revealed higher errors than males in the values calculated by the two equations compared to the direct measured HR_max_. Consequently, the study demonstrated that both equations tended to overestimate the HR_max_ for males and females over 15 years old, and the two universal equations were not suitable to predict the HR_max_ of Koreans except for children aged from 7 to 14 years. The new HR_max_ prediction equations suggested in this study will more accurately predict the HR_max_ of Asians, and additional analyses should be examined the cross-validity of the developed HR_max_ equation by age and gender in the future study.

## 1. Introduction

Maximal heart rate (HR_max_) is the highest heart rate of beats per minute measured by a maximal effort graded exercise test (GXT), and it is known as an important indicator for examining individuals’ cardio-respiratory fitness [1,2]. HR_max_ has been used as a basis for determining the upper limit of cardiovascular function and prescribing appropriate exercise intensity, along with other variables [3,4]. To prescribe effective and accurate exercise intensity, the American College of Sports Medicine (ACSM) has provided exercise prescription guidelines based on individuals’ HR_max_. For instance, the ACSM has recommended reaching 50~85% of an individual’s HR_max_ during exercise to improve aerobic fitness [5].

The most accurate method to measure HR_max_ is to be obtained by direct measure of HR at the highest exercise intensity during GXT [6]. Although it is difficult to evaluate whether the tested individuals have achieved maximal effort performance or near maximal effort performance, most previous studies used several physiological criteria to minimize this issue during GXT, such as subjective feeling (i.e., Rate of Perceived Exertion (RPE) > 17) and objective values (i.e., Respiratory Exchange Ratio (RER) > 1.10, post-exercise blood lactate levels ≥ 8 mmol·L^−^^1^, achievement of some percentage of an age-adjusted estimate of HR_max_, Plateau of ≤150 mL·O_2_·min^−1^ [7,8]). Nevertheless, even if GXT is the best way to measure the HR_max_, occasionally, it was not feasible or desirable to perform GXT because most participants were reluctant to suffer from fatigue induced during GXT. Therefore, when measuring the HR_max_ using GXT, we have to understand that recruiting participants for GXT is not only time-consuming but also costly [9].

Instead, one of the alternative methods for estimating HR_max_ is to predict it by using age-based regression equations. Several previous studies have demonstrated a decline in HR_max_ with increasing age, so individuals’ age has been used as a primary determinant for predicting HR_max_ [10], and using the age-based HR_max_ equations has become a common practice. Age-based formulas have been developed from a variety of studies [11,12,13,14,15,16], but perhaps the most commonly used equations are the Fox equation (HR_max_ = 220 − age) [17] and Tanaka equation (HR_max_ = 208 − 0.7 × age) [18] among the suggested equations. Fox equation has developed in 1971 and has been extensively investigated within the specific population (i.e., healthy, obese, and athlete) for adults [4,19,20], and especially, the HR_max_ prediction equation devised by Fox was widely utilized for physical activity and heart diseases study [21]. Later in 2001, the Tanaka equation was developed using a meta-analysis of 351 studies and showed high accuracy (r = −0.90) that there was no significant difference in gender, physical activity, sedentary behavior, or endurance-trained participants [22].

Although the abovementioned two equations (i.e., Fox and Tanaka) are ubiquitous equations for estimating HR_max_, some aspects need further research. A major limitation of these equations is that numerous studies for developing HR_max_ prediction equations and/or demonstrating validity recruited their participants as adults, especially males, in the last three decades [22,23]. Few studies have examined the pediatric population [3,24], but the studies had a common limitation on small sample sizes of less than 100 participants. Furthermore, Gulati’s study has found that the general age-based HR_max_ equations tended to be overestimated in females [13], and even the female athletes’ HR_max_ indicated significantly lower HR_max_ values compared to non-athletes [25]. Additionally, although several studies have revealed obvious differences in genetic factors that determine the physical indicators (i.e., height, weight, and body mass index) of Asians, Europeans, and/or Americans [26,27,28], most studies recruited Americans or Europeans for developing and/or validating HR_max_ formulas, so developed universal formulas may be difficult to predict the HR_max_ in Asians.

Therefore, the primary aim of the present study was to determine which equation (Fox and Tanaka) most accurately predicts HR_max_ in Korean compared to the direct measured HR_max_ using the large sample size of Asians. Additionally, the secondary aim was to focus on suggesting new equations for the Asian population by separating gender and age (i.e., 7–14 years, 15–24 years, 25–39 years, and 40–55 years [29]).

## 2. Methods

### 2.1. Study Protocol

The present study used the Bruce protocol and Metabolic Gas Analyzer Test System for the maximal GXT. Before the test, the metabolic gas analyzer was sufficiently operated, and the respiratory sensitivity transducer and gas concentration were checked. The measurement started at an initial speed of 1.7 mph and a grade of 10%, increasing the intensity every three minutes. Although all participants were encouraged to achieve maximum capability, the GXT was terminated when participants met at least three or more of the following five criteria: (1) increased exercise intensity but did not increase heart rate, (2) greater RER than 1.10, (3) higher RPE than 17 of Borg scale, (4) heart rate within ± 10 beats/min of age-predicted HR_max_ (Predicted HR_max_ was determined with the following equations of “220 − age” and “208 − 0.7 × age”), and (5) stagnant oxygen intake despite increased exercise intensity [2]. This study adopted the HR_max_ among the HR measured between the start and end of the GXT, and our study observed that participants’ HR did not exceed their HR_max_ for one minute immediately after GXT was terminated.

### 2.2. Study Participants

Table 1 presents a total of 672 participants aged from 7 to 55 years who were recruited for the study (male: 280 and female: 392). Participants aged from 7 to 14 years accounted for 30.21% (n = 203, 9.71 ± 2.11 years), aged from 15 to 24 was recruited by 23.21% (n = 156, 20.59 ± 2.35 years), aged from 25 to 39 indicated 22.62% (n = 152, 34.59 ± 4.57 years), and aged from 40 to 55 were 23.96% (n = 161, 43.71 ± 3.11 years). Before the test for the present study, we informed all participants regarding the study’s purpose, procedure, and possible occurrence of discomfort and risks. All participants aged above 18 years submitted written informed consent for participation in this study, and participants under the age of 18 years were asked to submit consent from each participant’s parent or legal guardian. All participants were aware that they could withdraw at any time without any prejudice. This study was approved by the Institutional Review Board (IRB) of Kyung Hee University (KHU IRB 2014-G06).

### 2.3. Measures

#### 2.3.1. Anthropometric Measurements

Body height (SECA S-208M, Chino, CA, USA) and weight (TANITA BC-581, Tokyo, Japan) were measured before the GXT began and were recorded in units of 0.1 cm and 0.1 kg, respectively. Additionally, body mass index (BMI) was calculated by dividing the weight (kg) by the square of the height (m^2^).

#### 2.3.2. Metabolic Gas Analyzer Test

Oxygen uptake was measured by Quark b^2^ (COSMED, Rome, Italy) during the GXT, which is a standard laboratory metabolic cart. The Quark b^2^ is a valid instrument to measure respiratory and metabolic parameters at the various intensity of exercise in the laboratory setting [30], and all participants completed the GXT test on the treadmill (Series 2000, Marquettem Electronic, Milwaukee, WI, USA) with the Bruce protocol [31].

#### 2.3.3. Maximal Heart Rate (HR_max_)

Heart rate was measured during the GXT using a wearable chest monitor and heart rate monitor (Polar RS400, Polar Electro Oy, Kempele, Finland). The Polar RS series was a chest strap, which in earlier studies has been demonstrated to have high validity and was well suited for measuring individuals’ heart rate during participation in physical activity and exercise training [32,33,34]. When participants reached at least three VO_2max_ criteria ((RPE > 17, RER > 1.10, achieved some percentage of an age-adjusted estimate of HR_max_, and Plateau of ≤ 150 mL·O_2_·min^−1^), the highest heart rate value was recorded as observed HR_max_.

### 2.4. Statistical Analysis

All data obtained from the present study were analyzed by SPSS 25.0 version (IBM SPSS, Chicago, IL, USA). Participants’ demographic information (i.e., gender, age, height, weight, and BMI) and maximal exercise response variables (i.e., RER, HR_max_, and VO_2max_) were summarized as mean and standard deviation (SD) by descriptive statistics. To compare with measured HR_max_ and the values calculated by the HR_max_ prediction equations (i.e., Fox equation and Tanaka equation), values were analyzed by one-way analysis of variance (ANOVA), two-way ANOVA (males × age categories, females × age categories), and Bonferroni post hoc test. Furthermore, to perform the cross-validation procedure, we utilized the following three statistical analysis methods (i.e., mean absolute percent errors, Bland–Altman plots, and equivalence testing) to confirm the consistency between the measured HR_max_ and the two prediction equations: (1) The Bland–Altman plot was performed with corresponding parameters (i.e., intercept and slope) to demonstrate the general agreement with corresponding ± 95% limit of agreement with fitted lines (from regression analyses between mean and difference). (2) Equivalence testing was performed to examine the equivalence between the measured HR_max_ and the predicted HR_max_. HR_max_ measured in each different method was demonstrated to be equivalent when the 90% confidence interval (CI) for the mean of the predicted HR_max_ was within the proposed equivalence limit (±10%) of the measured HR_max_. Lastly, the current study conducted multiple regression methods to develop HR_max_ prediction equations. (3) To estimate errors between the HR_max_ calculated by two equations (i.e., Fox and Tanaka′s equation) and direct measured HR_max_, Mean Absolute Percent Errors (MAPE) was used, which is a performance evaluation index that has been widely used to predict errors between the criterion method and other methods. In the simple regression, the dependent variable for the analysis was measured HR_max_, and the independent variable was age. We investigated each separate different equation and used a Monte Carlo cross-validation procedure.

## 3. Results

Table 2 presents the comparison of average HR_max_ divided by gender and age in each different HR_max_ estimation method (i.e., direct measurement, Fox′s equation, and Tanaka′s equation). The overall HR_max_ of participants aged from 7 to 55 was significantly different from Fox′s prediction equation (*p* < 0.001). However, the measured HR_max_ of male participants showed significant differences compared to those obtained by Tanaka′s prediction equation (*p* < 0.001), and the measured HR_max_ of females indicated significant differences from both equations (i.e., Fox′s equation and Tanaka′s equation) (*p* < 0.001). In addition, when the measured HR_max_ and the HR_max_ predicted by formulas (i.e., Fox′s equation and Tanaka′s equation) were compared by age, the HR_max_ predicted by Fox′s equation significantly differed from measured HR_max_ in all age groups (*p* < 0.001) except for the boys aged from 7 to 14 (*p* = 1.000), and the HR_max_ predicted by Tanaka equation revealed significant differences with measured HR_max_ in all ages (*p* < 0.01). Specifically, the measured HR_max_ in boys aged from 7 to 14 was a significant difference from the Tanaka prediction formula (*p* < 0.001), while the male adults aged from 40 to 55 indicated a significant difference from Fox′s prediction formula (*p* < 0.05). Additionally, the measured HR_max_ of youth in males aged from 15 to 24 was a significant difference from both equations (*p* < 0.001 and *p* < 0.01), but adults aged from 25 to 39 had no significant difference from the two equations (*p* = 1.000 and *p* = 1.000). In females, only the measured HR_max_ of children aged from 7 to 14 had no significant difference from Fox′s prediction formula (*p* = 1.000).

**Figure 1:**
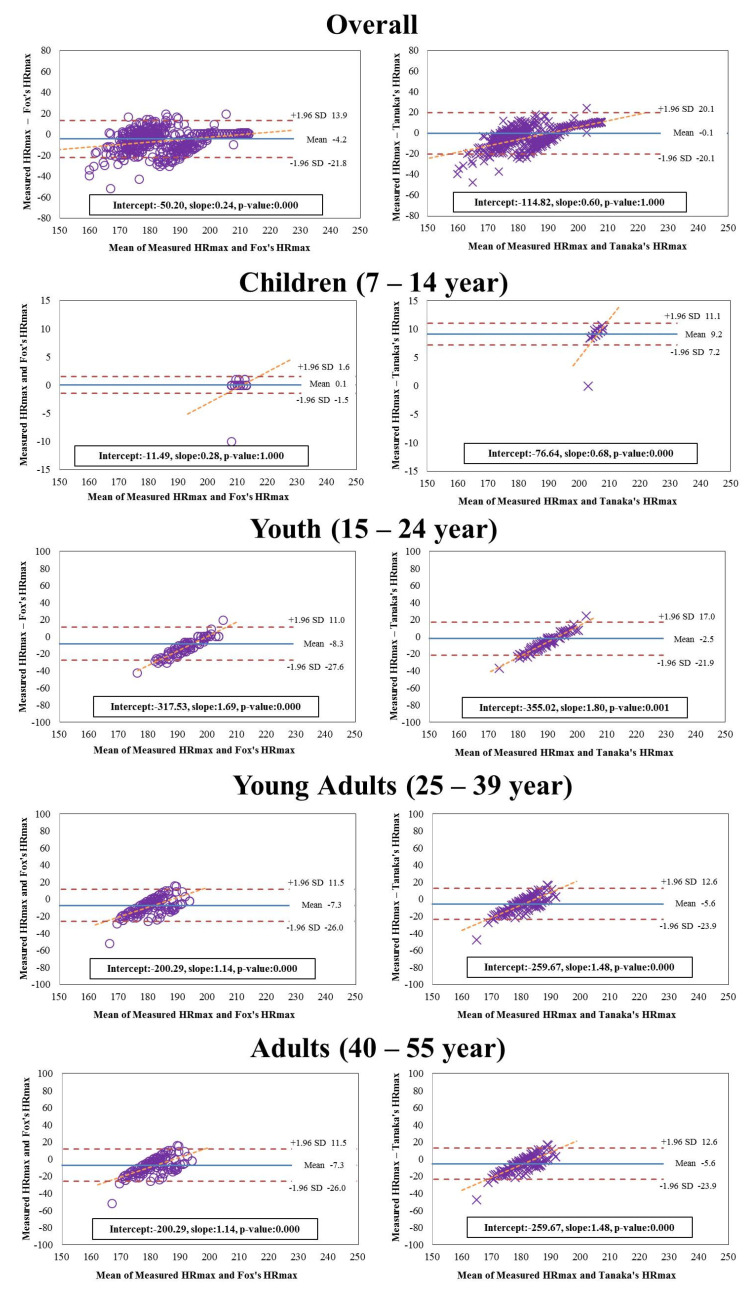
Bland–Altman plots for maximal heart rate estimated by Fox′s equation and Tanaka′s equation based on direct measured maximal heart rate.

Figure 1 illustrates the Bland–Altman plot, which is the extent of agreement between the criterion (i.e., directly measured HR_max_) and each equation (i.e., Fox′s equation and Tanaka′s equation) by using the mean difference values to demonstrate the proportional systematic bias, and the 95% limits of agreement and line of best fit were marked. The Tanaka′s HR_max_ prediction equation had the best fit in mean of the difference closer to zero (mean = −0.1, difference = 40.2) compared to the Fox′s HR_max_ prediction equation (mean = −4.2, difference = 35.7). However, there was a different outcome of fit in the agreement of children aged from 7 to 14, in which Fox′s prediction equation had the best fit in the average of the difference closer to zero (mean = 0.1, difference = 3.1). The slope of the fitted line tended to increase in the positive direction in all age groups, and the measured HR_max_ of participants aged 15 or older and the HR_max_ predicted by each equation showed a significant difference (*p* < 0.001).

**Figure 2:**
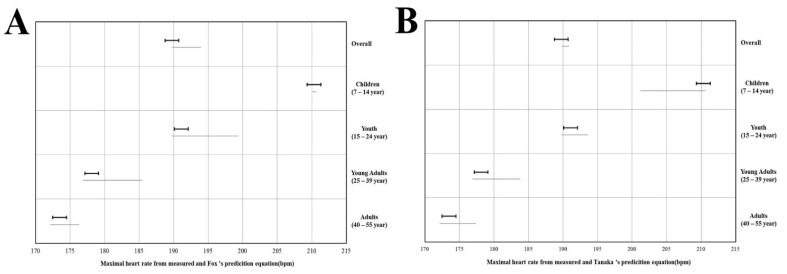
Dark lines mean equivalence zone (±10% of the mean). Grey lines are the 90% confidence interval for a mean of the estimated maximal heart rate prediction equation. (**A**) Equivalence testing in maximal heart rate measured by direct measurement and Fox′s equation; (**B**) equivalence testing in maximal heart rate measured by direct measurement and Tanaka′s equation.

Figure 2 shows the equivalence testing, and it can be examined whether each prediction equation estimates were equivalent to the criterion HR_max_. The HR_max_ evaluated by Fox′s prediction equation was completely included within the equivalence zone (±10% of the criterion measurement) only in children aged from 7 to 14, and all estimated values by Tanaka′s prediction equation were not fully included within the equivalence zone (±10% of the criterion measurement).

**Figure 3:**
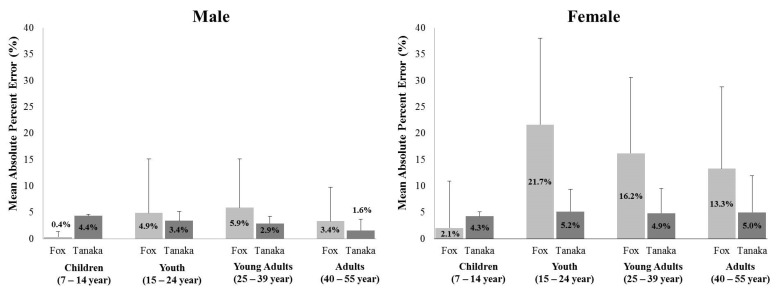
Mean absolute percentage error (MAPE) of maximal heart rate for Fox′s equation and Tanaka′s equation based on direct measured maximal heart rate.

Specifically, Figure 3 reveals the Mean Absolute Percent Errors (MAPE) calculated to investigate the difference between the criterion and the equations for gender groups (males, n = 280 and females, n = 392). For both males and females, the measured HR_max_ of children aged from 7 to 14 indicated a higher MAPE in Tanaka′s formula (males: 4.4% and females: 4.3%) than the HR_max_ predicted by Fox′s formula (males: 0.4% and females: 2.1%), and the HR_max_ predicted by Fox′s formula in participants aged 15 or older showed a higher MAPE. In particular, the MAPE of females over 15 years of age revealed higher MAPE than that of males in both the values calculated by Fox′s prediction equation and Tanaka′s prediction equation.

Table 3 indicated the regression coefficients for the measured HR_max_ equations. While, the overall HR_max_ equation in male was F = 2037.760 (*p* < 0.001, adj R^2^ = 0.880, SEE = 4.154, Durbin–Watson = 0.940), the overall HR_max_ equation in female was F = 355.193 (*p* < 0.001, adj R^2^ = 0.475, SEE = 10.319, Durbin–Watson = 1.726). To be specific, boys aged from 7 to 14 indicated F = 8443.111 (*p* < 0.001, adj R^2^ = 0.980, SEE = 0.311, Durbin–Watson = 1.087), but girls aged from 7 to 14 indicated F = 17.603 (*p* < 0.001, adj R^2^ = 0.364, SEE = 1.869, Durbin–Watson = 2.191). Furthermore, HR_max_ equation of youth aged from 15 to 24 in male had F = 6.376 (*p* < 0.001, adj R^2^ = 0.081, SEE = 6.914, Durbin–Watson = 0.636) and in female was F = 5.796 (*p* < 0.001, adj R^2^ = 0.049, SEE = 10.170, Durbin–Watson = 1.590). Both young male and female adults in male and in female aged from 25 to 39 revealed F = 3.456 (*p* < 0.001, adj R^2^ = 0.260, SEE = 6.462, Durbin–Watson = 1.844) and F = 2.748 (*p* < 0.001, adj R^2^ = 0.012, SEE = 9.146, Durbin–Watson = 1.884), respectively. Male adults aged from 40 to 55 showed F = 4.447 (*p* < 0.001, adj R^2^ = 0.087, SEE = 4.994, Durbin–Watson = 1.943) and female adults aged from 40 to 55 indicated F = 1.838 (*p* < 0.001, adj R^2^ = 0.007, SEE = 10.968, Durbin–Watson = 2.200).

## 4. Discussion

The current study demonstrated the validity of HR_max_ predicted by Fox′s formula and Tanaka′s formula based on direct measured HR_max_ and developed new HR_max_ prediction equations for gender and age (i.e., 7–14 years, 15–24 years, 25–39 years, and 40–55 years) by using direct measured HR_max_ as a criterion.

When predicting HR_max_ with Fox′s equation, the present study found significant differences in all gender and age groups except for boys and girls and only male young adults. The most noticeable finding was a significant difference in females′ HR_max_ predicted by Fox′s equation and the measured HR_max_. The reason is that since Fox′s HR_max_ prediction equation often has been applied to non-athletic males of a wide range of ages [35], females may have been underrepresented to predict HR_max_ based on the formula. Furthermore, the majority of research regarding Fox′s equation has reported that the equation had a standard deviation of about 7–13 bpm [2,20,21], which is consistent with the outcome overestimated approximately 9 bpm in the current study. This may not be suitable for predicting HR_max_ of the general population because Fox′s equation was determined based on a review of 10 studies without proper regression analysis and developed in older adults (over 60 years of age) with cardiovascular diseases. Even some studies have demonstrated equation had significantly over and/or underestimated HR_max_ in healthy younger and older adults [36,37,38], and our result was also consistent with the previous studies′ outcome by finding overestimating HR_max_. Although Fox′s equation may seem appropriate for boys and girls aged from 7 to 14 years, the present study suggests that Fox′s equation developed in older adult populations with cardiovascular diseases should be applied with caution to the general population, especially healthy people.

Tanaka′s equation, one of the HR_max_ prediction equations, seemed to be suitable for predicting HR_max_ of the general population aged from 7 to 55 years who participated in the present study (*p* > 0.05), which is similar to previous studies [39]. Unfortunately, Tanaka′s equation is difficult to predict for specific populations as an equation of age-based HR_max_. Unlike male adults over the age of 25, this study found that there were significant differences between HR_max_ measured directly and predicted by Tanaka′s equation in children (*p* < 0.001) and youth (*p* < 0.001). The finding might be because Tanaka′s equation was derived from adult populations aged from 20 to 81, excluding those younger than 20 years of age. In addition, HR_max_ predicted by Tanaka′s equation showed significant differences from the actual HR_max_ of females in all age groups, which indicated a discrepancy in the results of some existing literature [13,40]. Since the previous studies have investigated the validity of the existing formula on female athletes in America and/or Europe, and there were no studies that had developed the HR_max_ formula for females, an explanation of this discrepancy might be the different samples’ characteristics (i.e., general population and race). Therefore, our finding indicated that applying Tanaka′s equation to predict the HR_max_ of females needs to be careful and demonstrated that new equations for predicting the HR_max_ of females should be devised.

Overall, our study found significant differences between the direct measured HR_max_ and predicted HR_max_ by two equations in each different gender and most age groups, proving that it is not appropriate for the previously developed HR_max_ prediction equation to be applied equally to all age groups and each different gender. According to some studies, the fact that HR_max_ decreased with age may affect only adults more than children or adolescents [24,41,42]. However, the current study proved that HR_max_ in children, youth, and young adults had an association with age closely, but rather, HR_max_ in middle-aged adults over 40 years of age had a slightly low association with age compared to the other age groups. The finding might be suggested that not only age but also other factors such as gender, body fat percentage, and VO_2max_ may affect HR_max_ in middle-aged adults.

The present study has the following positive strengths and limitations. To the best of our knowledge, no other studies have developed HR_max_ prediction equations separately by gender and specific age groups in the Asian population despite the HR_max_ prediction equation having been developed in several studies. Furthermore, the main findings of this study proved the need to develop new equations through cross-validity based on existing formulas (i.e., Fox′s equation and Tanaka′s equation) and provided new insights into the HR_max_ prediction equation. However, since our study demonstrated the validity between HR_max_ predicted by two different equations compared to the direct measured HR_max_ and developed new equations, future studies need to verify the validity of the HR_max_ prediction equation regarding gender and age groups developed in the current study. Additionally, the participants in this study were limited to Korean participants. Therefore, it is necessary to examine more evidence from various ethnicities and adequate sample sizes from the same perspective.

## 5. Conclusions

The study proved that both Fox and Tanaka′s equations tended to overestimate the HR_max_ for males and females over 15 years old, and two universal equations were not suitable to predict the HR_max_ of Koreans except for children aged from 7 to 14. Since most studies have developed and examined HR_max_ for Americans or Europeans, the new HR_max_ prediction equations suggested in this study will more accurately predict the HR_max_ for Asians. Additional analyses should explore the cross-validity based on the HR_max_ prediction equation by gender and age presented in the study.

## Figures and Tables

**Table 1:** Participants’ characteristics (n = 672).

Variables			All Participants	
			No. (%)	Mean ± SD
Anthropometrics × Age	Height (cm)	7–14		144.22 ± 14.35
		15–24		167.69 ± 7.88
		25–39		162.54 ± 5.55
		40–55		163.45 ± 7.96
	Weight (kg)	7–14		39.62 ± 17.96
		15–24		62.66 ± 10.36
		25–39		59.01 ± 9.21
		40–55		64.35 ± 11.53
	BMI (kg·m^−2^)	7–14		18.14 ± 4.27
		15–24		22.13 ± 2.63
		25–39		22.27 ± 3.21
		40–55		23.92 ± 3.27
maximal exercise Responses × Age	HR_max_ (bpm)	7–14		209.89 ± 7.12
		15–24		201.12 ± 13.81
		25–39		193.64 ± 18.96
		40–55		189.90 ± 21.40
	VO_2max_ (mL·kg^−1^·min^−1^)	7–14		53.76 ± 9.20
		15–24		46.05 ± 12.30
		25–39		34.08 ± 6.45
		40–55		33.22 ± 7.21
	Respiratory exchange ratio	7–14		1.17 ± 0.13
		15–24		1.22 ± 0.14
		25–39		1.18 ± 0.08
		40–55		1.18 ± 0.09

SD—standard deviation, BMI—body mass index; HR_max_—maximal heart rate; VO_2max_—maximal oxygen uptake.

**Table 2:** Average of HR_max_ divided by gender, age, and gender × age in each different HR_max_ equation (Mean ± SD).

Variables			Measured HR_max_	Fox′s HR_max_	Tanaka′s HR_max_
Overall			189.77 ± 17.17	193.99 ± 13.74 ***	189.79 ± 9.67
Gender	Male		202.56 ± 11.96	202.98 ± 11.63	196.08 ± 8.14 ***
	Female		180.63 ± 14.24	187.57 ± 11.31 ***	185.29 ± 7.91 ***
Age (year)	7–14		210.35 ± 2.22	210.29 ± 2.11	201.20 ± 1.47 ***
	15–24		191.11 ± 10.34	199.41 ± 2.35 ***	193.59 ± 1.64 **
	25–39		178.15 ± 9.46	185.41 ± 4.57 ***	183.79 ± 3.20 ***
	40–55		173.49 ± 10.40	176.29 ± 3.11 ***	177.40 ± 2.18 ***
Gender × Age	Male	7–14	210.32 ± 2.20	210.21 ± 2.13	201.14 ± 1.49 ***
		15–24	196.76 ± 7.21	200.05 ± 2.64 ***	194.03 ± 1.85 **
		25–39	189.13 ± 7.51	190.25 ± 5.97	187.17 ± 4.17
		40–55	178.95 ± 5.22	176.86 ± 2.41 *	177.80 ± 1.69
	Female	7–14	210.57 ± 2.34	210.77 ± 1.87	201.53 ± 1.30 ***
		15–24	187.38 ± 10.42	198.99 ± 2.03 ***	193.29 ± 1.42 ***
		25–39	177.54 ± 9.20	185.15 ± 4.35 ***	183.60 ± 3.04 ***
		40–55	171.86 ± 11.05	176.11 ± 3.27 ***	177.27 ± 2.29 ***

SD—standard deviation; HR_max_—maximal heart rate. *** *p* < 0.001, ** *p* < 0.01, and * *p* < 0.05.

**Table 3:** Regression coefficients for estimating maximal heart rate.

Variables		Parameter	HR_max_ Regression Equation		New HR_max_ Prediction Equation
			Coefficients (95% CI)	Standard Error	
Overall	Male	Intercept	218.984 (218.117 to 219.851)	0.440	219 − age
		Age	−0.965 (−1.007 to −0.923)	0.021	
	Female	Intercept	208.830 (205.715 to 211.945)	1.584	209 − (0.9 × age)
		Age	−0.869 (−0.960 to −0.779)	0.046	
Children	Male	Intercept	220.310 (220.090 to 220.529)	0.111	220 − age
		Age	−1.020 (−1.042 to −0.998)	0.011	
	Female	Intercept	217.760 (214.179 to 221.340)	1.748	218 − (0.8 × age)
		Age	−0.779 (−1.159 to −0.399)	0.186	
Youth	Male	Intercept	213.619 (200.147 to 227.090)	6.735	214 − (0.8 × age)
		Age	−0.845 (−1.515 to −0.176)	0.335	
	Female	Intercept	213.600 (191.871 to 235.328)	10.940	214 − (1.2 × age)
		Age	−1.248 (−2.277 to −0.218)	0.518	
Young Adults	Male	Intercept	211.751 (181.448 to 242.053)	12.384	212 − (0.8 × age)
		Age	−0.761 (−1.762 to 0.241)	0.409	
	Female	Intercept	187.700 (175.492 to 199.907)	6.175	189 − (0.3 × age)
		Age	−0.291 (−0.639 to 0.056)	0.176	
Adults	Male	Intercept	210.269 (180.067 to 240.471)	14.877	210 − (0.7 × age)
		Age	−0.726 (−1.425 to −0.027)	0.344	
	Female	Intercept	189.836 (163.521 to 216.150)	13.293	190 − (0.4 × age)
		Age	−0.410 (−1.007 to 0.188)	0.302	

SD—standard deviation; CI—confidence interval; HR_max_—maximal heart rate; Overall—from 7 to 55 years old; Children—from 7 to 14 years old; Youth—from 15 to 24 years old; Young Adults—from 25 to 39 years old; Adults—from 40 to 55 years old.

## Data Availability

The datasets used and/or analyzed during the current study are available from the corresponding author on reasonable request.
